# ﻿*Primulaxilingensis* (Primulaceae), a new species from Sichuan, China

**DOI:** 10.3897/phytokeys.234.108411

**Published:** 2023-10-18

**Authors:** Junjia Luo, Mingke Zhang, Xiaofeng Liu, Hui Chen, Tingyu Li, Xudong Ma, Ke Huang, Zhixi Fu

**Affiliations:** 1 Key Laboratory of Land Resources Evaluation and Monitoring in Southwest, Sichuan Normal University, Ministry of Education, Chengdu 610066, China Sichuan Normal University Chengdu China; 2 College of Life Sciences, Sichuan Normal University, Chengdu 610101, China Sichuan Normal University Chengdu China; 3 Sustainable Development Research Center of Resources and Environment of Western Sichuan, Sichuan Normal University, Chengdu 610066, China Sichuan Normal University Chengdu China

**Keywords:** conservation, morphological characters, *Primula* sect. *Minutissimae*, taxonomy

## Abstract

A new species, *Primulaxilingensis* K.Huang & Z.X.Fu, **sp. nov.** (Primulaceae), is described and illustrated. In gross morphology, it is clearly allied to section Minutissimae on account of having stolons, being glabrous, leaf rosette less than or equal to corolla, flower solitary and bract not swollen at base. The new species is easily distinguished by the combination of scape densely yellow farinose, leaf apex acute, rarely broadly obtuse, corolla pale purplish blue and style 3.0–6.0 mm above base of corolla tube, stamens reaching the corolla tube mouth in thrum flower. In addition, the distribution map, morphological comparison of related species and conservation status of the new species are also provided.

## ﻿Introduction

The genus *Primula* L. is one of the largest genera in the Primulaceae, encompassing approximately 536 species (Ju et al. 2023; Li et al. 2023; POWO 2023; Wang et al. 2023; Wu et al. 2023a, b; Yang et al. 2023; Zhang et al. 2023). In China, the last comprehensive account of the genus was that of Hu and Kelso (1996) who treated 300 species in the “Flora of China”. The additional several new species of *Primula* were added by e.g., Li and Hu (2009; one species), Xu et al. (2016a, b, 2017, 2022; four species), Wu et al. (2019; one species), Ju et al. (2021; one species), Wang et al. (2022; one species), Li et al. (2023; one species), and Yang et al. (2023; two species), bringing the total number of species in China to *c.* 340 species.

In China, Primulasect.Minutissimae Pax has approximately 17 species, and is abundantly distributed to Xizang, Yunnan and Sichuan provinces (Hu and Kelso 1996). This section can be distinguished by a set of morphological characters, in which members are dwarf perennial herbs, glabrous or with glandular hairs, often with stolons, leaf rosette less than or slightly larger than corolla, bracts small and not swollen at base, flower usually solitary, rarely 2–4, and capsule nearly as long as calyx (Hu 1990; Hu and Kelso 1996).

During a botanical expedition led by the last author to Xiling Snow Mountain, Dayi city, Sichuan Province in May 2022, a population of *Primula* was discovered, photographed and collected. Based on flowering taxon photos, the taxon appears to be closely related to *Primulatenella* King ex G.Watt. and *Primuladujiangyanensis* W.B.Ju, Bo Xu & X.F.Gao. After consultation with relevant literature and morphological examination of closely related taxa, it proved that it is eventually represents an unreported taxon of P.sect.Minutissimae. The new species can be differentiated from other members of this section by the combination of corolla pale purplish blue, scape densely yellow farinose with a solitary flower, and the style 3.0–6.0 mm above base of corolla tube, and stamens reaching the corolla tube mouth in thrum flower. Thus, it is described and illustrated as new to science below.

## ﻿Material and methods

The observation and collection of both herbarium and living materials of the new species from Xiling Snow Mountain, Dayi city, Sichuan Province were conducted in May 2022 and June 2023. Morphological comparison of taxonomic literature of the closely related species, i.e. *P.dujiangyanensis* (Ju et al. 2021, herbarium CDBI, holotype, *DJY00272*), *Primulapengzhouensis* C.M.Hu, G.Hao & Y.Xu (Xu et al. 2017, herbarium IBSC, holotype, *Xu16009*) and morphological comparison of specimens’ images from Global Plants JSTOR (https://plants.jstor.org), i.e. *P.tenella* (herbarium K, holotype, *K000639442*, photo!, herbarium E, isotype, *E00024523*, photo!) were consulted. Morphological description and measurements of *P.xilingensis* were based on living plants. The taxonomic description follows the terminology used by Beentje (2016). The type and voucher specimens are stored at herbarium SCNU (follows Thiers 2023). The conservation status of the new species was assessed following the guidelines of the IUCN Red List categories and criteria (IUCN 2022).

## ﻿Taxonomic treatment

### ﻿ Primula xilingensis

Taxon classificationPlantaeEricalesPrimulaceae

K.Huang & Z.X.Fu sp. nov

BB7C373A-2771-5C9B-9521-023C0449D77C

urn:lsid:ipni.org:names:77328946-1

#### Diagnosis.

**Figure 1.**
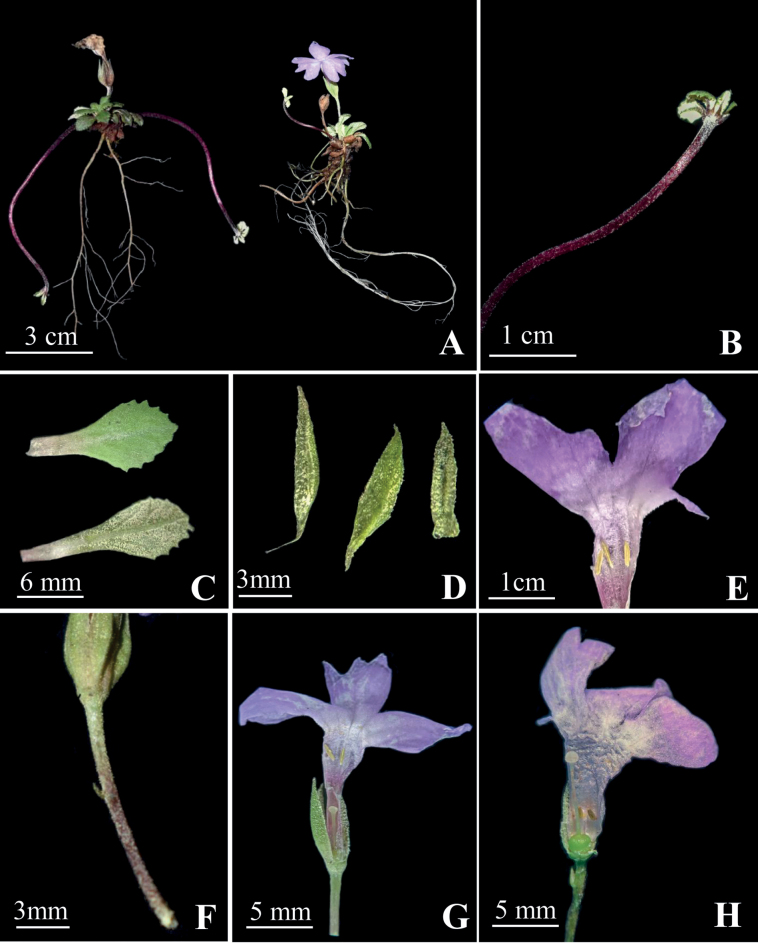
*Primulaxilingensis* sp. nov. **A** plant and roots **B** stolon **C** leaves **D** calyx **E** stamens **F** scape and bract **G** thrum flower **H** pin flower. (Photos **A, B** by XM, and **C–H** by MZ).

**Figure 2.**
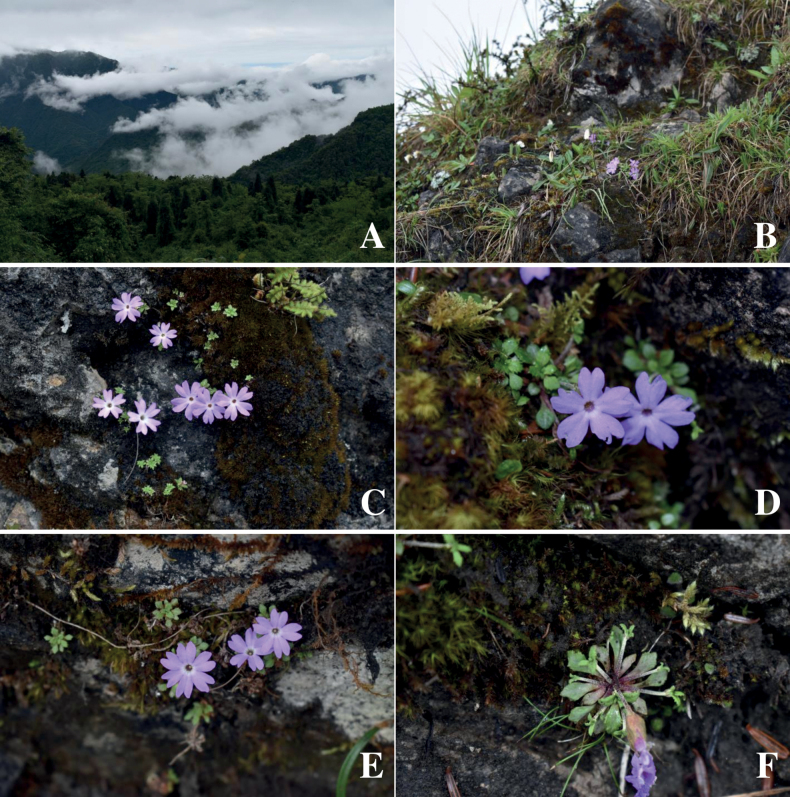
*Primulaxilingensis* sp. nov. **A, B** habitat **C** plants of thrum flower **D** habit **E** plants of pin flower **F** stolons. (Photos **A, B** by ZF, **C, D** by KH, and **E, F** by MZ).

**Figure 3.**
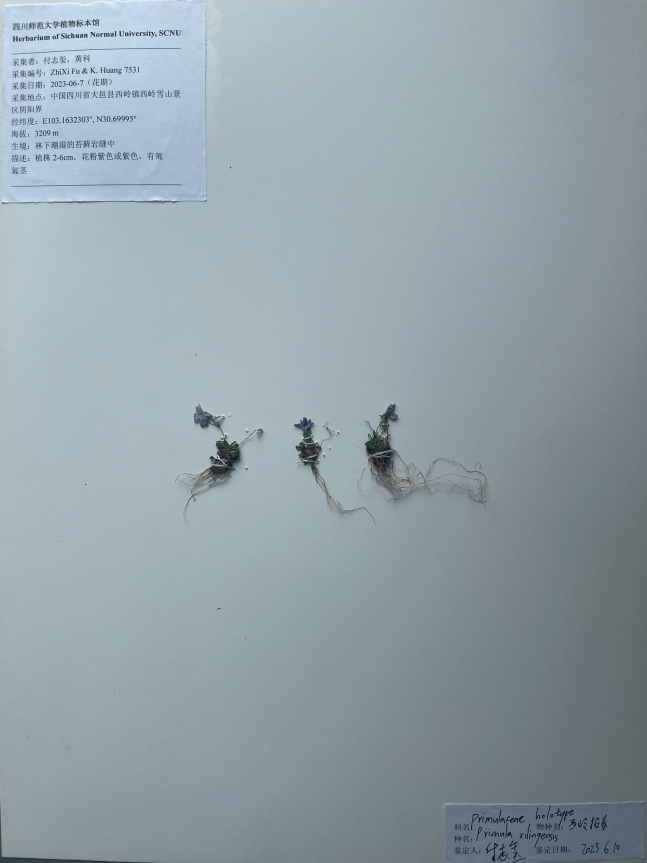
Holotype image of *Primulaxilingensis* K.Huang & Z.X.Fu, sp. nov.

The new species is easily recognised by the following combination of characters: the scape densely yellow farinose, leaf apex acute, rarely broadly obtuse, corolla pale purplish blue and the style 3.0–6.0 mm above base of corolla tube, and stamens reaching the corolla tube mouth in thrum flower (Figs 1–3).

#### Type.

China. Sichuan: Dayi City, Xiling Snow Mountain, growing on moist rock surfaces amidst moss under the forest. 30°41′59.82″N, 103°9′47.63″E, alt. *c.* 3200m, 7^th^ June 2023 (fl.), *K. Huang & Zhixi Fu 7531* (holotype, SCNU!) (Figs 1–3).

#### Description.

A perennial herb, 1.0–4.0 cm tall. **Stolons** dark red, 2.0–6.0 cm long developing onwards leafless flagellate, each with a tiny rosette at the end, and clothed the base by the withered remains of the old leaves. ***Roots*** numerous, fibrous, without hairs, 3.0–12.0 cm long. ***Leaves*** in a loose to tight rosette, 1.0–2.0 cm in diameter, leaf rosette less than or equal to corolla; leaf blade ovate to ovate–elliptic, 5.0–8.0 × 3.0–6.0 mm, margin dentate generally in the upper half only, apex acute, rarely broadly obtuse, tapering to base forming a winged petiole, petioles usually shorter than leaf blade and sparingly farinose, densely yellow farinose abaxially, midvein prominent, sparingly yellow farinose adaxially, veins inconspicuous. ***Scape*** solitary, 0.2–1.5 cm tall, erect, densely yellow farinose, usually with a single flower. ***Bracts*** solitary, 1.0–2.0 mm long, lanceolate, not swollen at base. ***Pedicel*** slightly bent, densely yellow farinose. ***Flowers*** heterostylous. ***Calyx*** narrowly campanulate, prominent 5-veined, 3–9 mm long, densely yellow farinose, parted to 2/3 of its length or slightly below, lobes linear lanceolate to lanceolate, apex acute. ***Corolla*** pale purplish blue, lacking the appendage on the corolla tube, 5 lobes spreading, obovate, 4.0–8.0 mm long, densely yellow farinose abaxially, efarinose and glabrous adaxially, deeply emarginate. ***Pin flower***: corolla tubes 5.0–10.0 mm long in length, 2–3 mm in diameter, longer than calyx, stamens 2.0–5.0 mm above base of corolla tube, style nearly as long as tube. ***Thrum flower***: corolla tubes 7.0–13.0 mm long in length, *c.* 3 mm in diam, longer than calyx, style 3.0–6.0 mm above base of corolla tube, stamens reaching the corolla tube mouth (Figs 1–3). ***Capsule*** unknown.

#### Phenology.

Flowers collected in May and June.

#### Etymology.

The epithet “*xilingensis*” is derived from Xiling Snow Mountain, the snow mountain located in Dayi City, Sichuan Province, China.

#### Distribution and habitat.

**Map 1.**
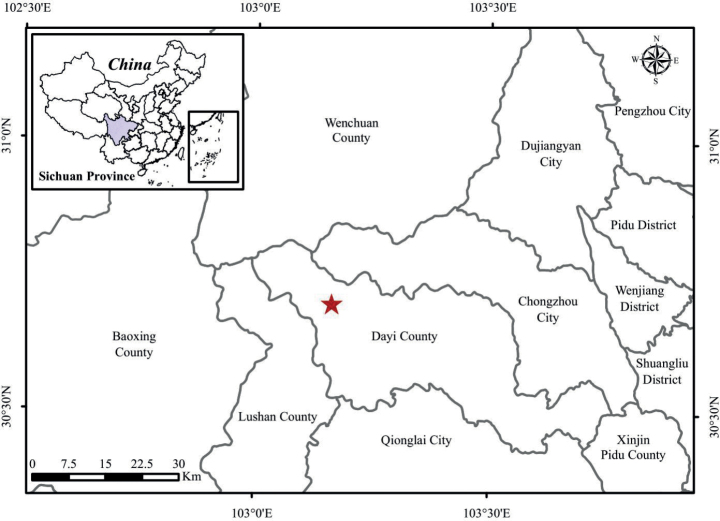
Location of the population of *Primulaxilingensis* in Dayi County, Sichuan (red star).

*P.xilingensis* is currently known only from its type locality in Yingyangjie, Xiling Snow Mountain, Xiling Town, Dayi City, Sichuan Province, China (Map 1). It grows on moist rock surfaces amidst moss under the forest, at elevations of approximately 3200 m (Fig. 2).

#### Additional specimens examined

**(paratypes).** CHINA. Sichuan: Dayi City, Xiling Snow Mountain, 30°41′59.82″N, 103°9′47.63″E, 21 May 2022 (fl.), *Zhixi Fu 6052* (SCNU!); ibid., 7 June 2023 (fl.), *K. Huang & Zhixi Fu 7532 & 7533* (SCNU!).

#### Conservation status.

Data Deficient (DD). Currently, only one population with more than 100 individuals has been found in the type locality. The population of *P.xilingensis* grows on moist rock surfaces amidst moss, and the Xiling Snow Mountain has corresponding protective measures for the environment and plants. Whilst currently only known from Xiling Snow Mountain, it is very possible that the taxon is found in other localities and, without a more comprehensive collecting programme, and known only from two collections, it would be best to assess the conservation status of the species as Data Deficient (DD) following the IUCN Red List criteria (IUCN 2022).

#### Relationship with related species.

Critical examination of collected specimens, comparison with type material of allied taxa, and relevant taxonomic literature revealed that *P.xilingensis* is a new member of the P.sect.Minutissimae. Morphologically, *P.xilingensis* shares certain similarities with *P.dujiangyanensis*. However, *P.xilingensis* differs from *P.dujiangyanensis* in having the style nearly as long as tube in pin flowers (vs. the style slightly exceeding the corolla tube mouth in pin flowers), stamens reaching the corolla tube mouth in thrum flowers (vs. stamens inserted on the apex of the corolla tube, scarcely exceeding 1 mm in thrum flowers), lacking an appendage on the corolla tube (vs. a yellow appendage present on the corolla tube), having numerous roots without hairs (vs. few roots with dense white hairs), corolla pale purplish blue (vs. corolla pinkish purple), leaf apex acute (vs. leaf apex broadly obtuse to sub-rounded) and distributed at about 3200 m elevation (vs. 1550–1650 m). To some extent, it also resembles *P.tenella* as a perennial herb with stolons, farinose leaves, margin dentate generally on the upper half only, but differs from *P.tenella* in leaf blade (ovate to ovate-elliptic vs. rhomboid to ovate-spatulate), leaf size (5.0–8.0 × 3.0–6.0 mm vs. 6.0–15.0 × 5.0–8.0 mm), leaf farinose (yellow vs. white) and altitude (at an altitude of approximately 3200 m vs. 4700–5000 m). The species of *P.xilingensis* is similar to *P.pengzhouensis*, but can be easily distinguished from the former by its stolons (present with 2–6 cm long vs. absent), scape visibility (vs. almost obsolete) and altitude (*c.* 3200 m vs. *c.* 1170 m). Further morphological comparisons among the species of *P.xilingensis*, *P.dujiangyanensis*, *P.pengzhouensis*, and *P.tenella* are shown in Table 1.

**Table 1.** Morphological characters comparison between *P.xilingensis* and closely related species of *P.dujiangyanensis*, *P.pengzhouensis* and *P.tenella*.

Features	*P.xilingensis*	*P.dujiangyanensis*	*P.pengzhouensis*	*P.tenella*
Roots	numerous, without hairs	few, dense white hairs	unknown	unknown
Stolon	2.0–6.0 cm long	3.0–6.0 cm long	absent	short leafy stolons
Leaf blade	ovate to ovate-elliptic, 5.0–8.0 × 3.0–6.0 mm	ovate to ovate-elliptic, 4.0–8.0 × 3.0–5.0 mm	elliptic to ovate elliptic, 2.0–3.5 × 1.5–2.2 cm	rhomboid to ovate-spatulate, 6.0–15.0 × 5.0–8.0 mm
Leaf farinose	abaxially densely yellow farinose, adaxially sparingly yellow farinose	abaxially copiously yellow farinose, adaxially sparingly yellow farinose	abaxially more or less covered with a fugacious yellow farina, adaxially efarinose	abaxially copiously white farinose, adaxially densely glandular and sparingly white farinose
Leaf apex	**acute, rarely broadly obtuse**	broadly obtuse to sub-rounded	broadly obtuse to sub-rounded	subrounded, rarely acute
Leaf margin	margin dentate generally in the upper half only	margin dentate or crenulate generally in the upper half only	margin serrate–dentate	margin usually denticulate or crenulate above middle
Scape	scape 1, 0.2–1.5 cm tall, usually with a single flower	scape 1, 1.0–2.5 cm tall, usually with a single flower, very rarely two	scape almost obsolete, at most 2.0 mm tall, bearing one terminal flower.	Scape 1, 2.0–5.0 cm tall, bearing 1(or rarely 2)-flowered
Scape farinose	**densely yellow farinose**	scarcely farinose	unknown	scarcely farinose
Calyx	narrowly campanulate,	narrowly campanulate	narrowly campanulate	narrowly campanulate
Calyx farinose	densely yellow farinose	sparingly yellow farinose outside, densely so inside	sparingly yellow farinose outside, parted slightly beyond middle	glandular outside, copiously white farinose inside
Corolla	**pale purplish blue**	pinkish purple	corolla rose or pale purple	corolla blue-violet
Corolla farinose	abaxially densely yellow farinose	unknown	sprinkled with yellow farina outside	unknown
An appendage on the corolla tube or not	absent	yellow appendage	absent	white appendage
Pin flowers	stamens 2.0–5.0 mm above base of corolla tube, style nearly as long as tube	stamens in the middle of corolla tube; style slightly exceeding the corolla tube mouth	stamens *c.* 3 mm above base of corolla tube, style *c.* 2/3 as long as corolla tube	stamens *c.* 3 mm above base of corolla tube, style reaching mouth
Thrum flowers	stamens reaching the corolla tube mouth, style 3.0–6.0 mm above base of corolla tube	stamens inserted on the apex of the corolla tube, scarcely exceeding 1 mm; style in the middle of the corolla tube	stamens up to 2/3 of corolla tube; style *c.* 3 mm	stamens at middle of corolla tube; style *c.* 3 mm
Altitude and distribution area	*c.* 3200 m, Dayi City, Sichuan Province	1550–1650 m, Dujiangyan City, Sichuan Province	*c.* 1170 m, Pengzhou City, Sichuan Province	4700–5000 m, Sounthern of Xizang Province

## Supplementary Material

XML Treatment for Primula xilingensis

## ﻿Additional information

### Author ORCIDs

Junjia Luo https://orcid.org/0009-0003-6453-9507

Mingke Zhang https://orcid.org/0009-0007-2494-7695

Xiaofeng Liu https://orcid.org/0009-0003-2471-6935

Hui Chen https://orcid.org/0009-0003-4786-7453

Tingyu Li https://orcid.org/0009-0005-6873-2114

Xudong Ma https://orcid.org/0009-0006-6303-0006

Ke Huang https://orcid.org/0009-0006-2057-5452

Zhixi Fu https://orcid.org/0000-0002-2789-6287

### Data availability

All of the data that support the findings of this study are available in the main text or Supplementary Information.

## References

[B1] Beentje H (2016) The Kew Plant Glossary, an Illustrated Dictionary of Plant Terms. Kew Publishing, 184 pp.

[B2] Hu CM (1990) *Primula*. In: Chen FH, Hu CM (Eds) Flora Reipublicae Popularis Sinicae (Vol. 59(2)). Science Press, Beijing, 277 pp.

[B3] Hu CM Kelso S (1996) Primulaceae. In: Wu ZY Raven PH (Eds) Flora of China (Vol.15). Science Press, Beijing & Missouri Botanical Garden Press, St. Louis, 99–185.

[B4] IUCN (2022) Guidelines for Using the IUCN Red List Categories and Criteria. Version 15. Prepared by the Standards and Petitions Committee.

[B5] Ju WB Deng HN Zhu DH Gao YD Gao XF Xu B (2021) *Primuladujiangyanensis* (Primulaceae) discovered from Sichuan, Southwest China. Phytotaxa 510(3): 275–280. 10.11646/phytotaxa.510.3.7

[B6] Ju WB Deng HN Liu F He XJ Gao XF Xu B (2023) *Primulamedogensis*, a new species of Primulaceae from Tibet of China. PhytoKeys 230: 107–114. 10.3897/phytokeys.230.107008 37576131 PMC10422595

[B7] Li R Hu CM (2009) *Primulalihengiana* (Primulaceae), a new species from Yunnan, China. Annales Botanici Fennici 46(2): 130–132. 10.5735/085.046.0208

[B8] Li X Cheng YH Lin HQ Chen C Gao XF Deng HN Yu F Anđelka PM Ju WB Xu B (2023) *Primulawolongensis* (Primulaceae), a new species of the primrose from Sichuan, China. PhytoKeys 218: 47–57. 10.3897/phytokeys.218.91161 36762277 PMC9846278

[B9] POWO (2023) Plants of the World Online. Facilitated by the Royal Botanic Gardens, Kew. http://www.plantsoftheworldonline.org/ [Retrieved 09 February 2023]

[B10] Thiers B (2023) Index Herbariorum: a global directory of public herbaria and associated staff. New York Botanical Garden’s Virtual Herbarium. http://sweetgum.nybg.org/science/ih [Retrieved 11 May 2023]

[B11] Wang ZH Wang Y Chen L Peng H Wu ZK Guo G (2022) *Primulalongipilosa* (Primulaceae), a new species from Yunnan, China. PhytoKeys 194: 15–22. 10.3897/phytokeys.194.81335 35586322 PMC9016029

[B12] Wang SQ Yan HF Cheng ZJ Wang YB (2023) *Primulaxingshanensis* (Primulaceae), a new species from Hubei, China. Phytotaxa 594(2): 158–162. 10.11646/phytotaxa.594.2.8

[B13] Wu ZK Zhao FW Chen JH Huang Y (2019) *Primuladongchuanensis* (Primulaceae), a new species from northern Yunnan, China. PhytoKeys 130: 171–181. 10.3897/phytokeys.130.35047 31534405 PMC6728386

[B14] Wu Y Yang WH Wu ZK (2023a) *Primulajiaozishanensis* (Primulaceae), a new species in Primulasect.Petiolaressubsect.Davidii from Yunnan, China. PhytoKeys 227: 25–33. 10.3897/phytokeys.227.103985 37287937 PMC10242401

[B15] Wu ZK Guo YJ Zhang T Burgess KS Zhou W (2023b) *Primulaluquanensis* sp. nov. (Primulaceae), a new species from southwestern China, reveals a novel floral form in the heterostyly-prevailing genus. Plants 12(3): e534. 10.3390/plants12030534 PMC9918951 36771618

[B16] Xu Y Li CH Hu CM Hao G (2016a) *Primulawawushanica* sp. nov. (Primulaceae) from Sichuan, southwestern China. Nordic Journal of Botany 34(2): 156–158. 10.1111/njb.00894

[B17] Xu Y Yu XL Hu CM Hao G (2016b) Morphological and molecular phylogenetic data reveal a new species of *Primula* (Primulaceae) from Hunan, China. PLoS ONE 11(10): e0165355. 10.1371/journal.pone.0165355 PMC5007043 27579832

[B18] Xu Y Huang G Hu C Hao G (2017) *Primulapengzhouensis* (Primulaceae), a new species from Sichuan, southwestern China. Plant Diversity 39(4): 229–231. 10.1016/j.pld.2017.08.003 30159516 PMC6112288

[B19] Xu Y He DM Yang LZ Hao G (2022) *Primulasurculosa* (Primulaceae), a new species from Yunnan, China. PhytoKeys 212: 29–35. 10.3897/phytokeys.212.91133 36761304 PMC9836474

[B20] Yang B Ya JD Zhang W Song Y Wang W Zhu ZM He JH Zuo YJ Tan YH (2023) Two new species of *Primula* (Primulaceae) from Yunnan, China. Taiwania 68(2): 230–240. 10.6165/tai.2023.68.230

[B21] Zhang N Jiang XQ Wu ZK (2023) *Primulapingbaensis* (Primulaceae), a new species from Guizhou, China. PhytoKeys 221: 85–93. 10.3897/phytokeys.221.97948 37250357 PMC10209618

